# The cerebellar cognitive affective syndrome scale reveals early neuropsychological deficits in SCA3 patients

**DOI:** 10.1007/s00415-021-10516-7

**Published:** 2021-03-20

**Authors:** Roderick P. P. W. M. Maas, Sven Killaars, Bart P. C. van de Warrenburg, Dennis J. L. G. Schutter

**Affiliations:** 1 Department of Neurology, Donders Institute for Brain, Cognition, and Behaviour, Radboud University Medical Center, Nijmegen, The Netherlands; 2 Experimental Psychology, Helmholtz Institute, Utrecht University, Utrecht, The Netherlands

**Keywords:** Cerebellar cognitive affective syndrome, Spinocerebellar ataxia type 3, Cerebellum, Cognition

## Abstract

**Background:**

The cerebellar cognitive affective syndrome scale (CCAS-S) was recently developed to detect specific neuropsychological deficits in patients with cerebellar diseases in an expedited manner.

**Objectives:**

To evaluate the discriminative ability of the CCAS-S in an etiologically homogeneous cohort of spinocerebellar ataxia type 3 (SCA3) patients and to examine relationships between cognitive deficits and motor symptom severity.

**Methods:**

The CCAS-S was administered to twenty mildly to moderately affected SCA3 patients and eighteen healthy controls matched for age, sex, and educational level. Disease severity was measured by the Scale for the Assessment and Rating of Ataxia (SARA), Inventory of Non-Ataxia Signs (INAS), 8 m walk test, nine-hole peg test (9HPT), and Patient Health Questionnaire-9 (PHQ-9).

**Results:**

SCA3 patients had a lower total CCAS-S score (*p* < 0.001) and higher number of failed tests (*p* = 0.006) than healthy controls. Patients displayed impairments in semantic fluency, phonemic fluency, category switching, cube drawing, and affect regulation. Total CCAS-S score showed high discriminative ability (area under the curve [AUC]: 0.96) and was associated with disease duration, SARA score, walking speed, and dominant hand 9HPT performance. No correlations were observed with INAS count, repeat length, and PHQ-9 score. Discriminative capacity of the number of failed tests was moderate (AUC: 0.76).

**Conclusion:**

Essentially all SCA3 patients exhibited some form of cognitive impairment. The CCAS-S differentiates SCA3 patients from healthy controls, detects neuropsychological deficits early in the disease course, and correlates with relevant ataxia severity measures.

## Introduction

Contrary to long-standing assumptions, converging evidence from multiple lines of research indicates a substantial cerebellar contribution to cognitive and affective processes [1–8]. Reciprocal connections between the cerebellum and prefrontal, posterior parietal, superior temporal, and paralimbic regions have been well established and provide an anatomical framework for cerebellar involvement in higher-order cognitive networks [9, 10]. Disruption of this elaborate cerebrocerebellar circuitry is currently considered the principal mechanism associated with the emergence of cognitive and emotional deficits in patients with cerebellar disorders [6, 11]. Indeed, impairments in executive functions, linguistic processing, visuospatial cognition, and affect regulation have repeatedly been delineated in individuals with degenerative cerebellar diseases and focal lesions confined to the posterior lobe [6, 12–14]. This constellation of symptoms and signs defines the cerebellar cognitive affective syndrome (CCAS) or Schmahmann’s syndrome.

Spinocerebellar ataxia type 3 (SCA3), also known as Machado–Joseph disease, is the most common form of dominantly inherited ataxia. In addition to the characteristic motor syndrome, the SCA3 phenotype frequently comprises non-motor features, including cognitive dysfunction [15]. Deficits in verbal fluency, executive functions, and visuospatial abilities have been reported, along with disturbances in other domains, such as verbal memory [16–22].

Because of the absence of a sufficiently sensitive screening instrument for the detection of CCAS, such as the Mini-Mental State Examination or Montreal Cognitive Assessment, extensive neuropsychological test batteries are typically required in patients with cerebellar ataxia [14]. Recently, a bedside tool with an administration time of approximately 15 min has been developed for this purpose that consists of ten sensitive tasks, selected out of a comprehensive neuropsychological assessment [14]. The CCAS/Schmahmann syndrome scale (CCAS-S) was validated and found to perform reasonably well in 39 individuals with 27 different cerebellar disorders and a significantly younger control group. Replication of these findings in independent, etiologically homogeneous groups of ataxia patients is imperative because it would further support the use of this instrument for screening and possibly follow-up purposes. The objectives of the present study were 1) to examine if the CCAS-S reliably discriminates between mildly to moderately affected SCA3 patients and healthy controls, 2) to determine whether cognitive dysfunction is associated with measures of motor symptom severity, and 3) to search for predictors of cognitive deficits.

## Methods

### Participants

Twenty adult SCA3 mutation carriers with clinically manifest ataxia and eighteen healthy controls who were matched for age, sex, and educational level volunteered to participate in this cross-sectional study. Patients were recruited from our ataxia outpatient clinic and through the Dutch ADCA/ataxia society between October 2018 and April 2019. Clinically manifest disease was defined by a Scale for the Assessment and Rating of Ataxia (SARA) score of three or more [23]. The SARA contains eight items, which together produce a score between 0 (no ataxia) and 40 (severe ataxia) [24]. Patients were mildly to moderately affected, as indicated by a mean SARA score of 11.9 (range 5.0–21.5). Healthy volunteers did not have a medical history of neurological, psychiatric, or oncological disease, and were not taking centrally acting medication, recreational drugs, or more than two drinks of alcohol per day. All participants were native Dutch speakers.

The study was approved by the local medical ethics committee (CMO region Arnhem-Nijmegen) and written informed consent was obtained from all participants.

### CCAS-S

The original version (1A) of the CCAS-S was translated into Dutch [25]. Administration of this version occurred in a quiet room by the same examiner at a similar time of day for each participant. The scale contains the following ten tasks: semantic fluency (language, animal category; maximum score 26 points, cut-off 15 points), phonemic fluency (language, words that start with the letter F; maximum score 19 points, cut-off 9 points), category switching (executive functions—cognitive flexibility; maximum score 15 points, cut-off 9 points), digit span forward (attention and working memory; maximum score 8 points, cut-off 5 points), digit span backward (working memory; maximum score 6 points, cut-off 3 points), cube draw and copy (visuospatial and visuoconstructive abilities; maximum score 15 points, cut-off 11 points), delayed verbal recall (verbal long-term memory; maximum score 15 points, cut-off 10 points), similarities (executive functions—abstract reasoning; maximum score 8 points, cut-off 6 points), go/no-go (executive functions—response inhibition; maximum score 2 points, cut-off 0 points), and affect regulation (maximum score 6 points, cut-off 4 points) [14]. Unlike the other nine tasks, which have objective scoring criteria, affect regulation is—by default—scored in a qualitative manner through clinical judgement by the examiner and observations from family members. Immediate verbal recall is also included in the CCAS-S but does not receive a formal score. Of these ten tests, only cube drawing and copying poses some demands on upper limb function. Within the design of the scale, the influence of motor symptoms on cognitive performance is therefore kept to a minimum.

Raw scores for each individual task were compared with previously assigned threshold values, as listed above, to determine if a participant passed or failed the item [14]. In addition to a raw score per test, the CCAS-S yields a sum score between 0 and 120 and a total number of failures between 0 and 10. Possible, probable, and definite CCAS were defined in the original paper as one, two, and three or more failed tests, respectively [14].

### Measures of disease severity

Administration of the CCAS-S was directly followed by a comprehensive clinical evaluation of ataxia severity and extracerebellar involvement. A movement disorders specialist with experience in the field of ataxia (BvdW) determined videotaped SARA scores in all SCA3 patients, while being unaware of their cognitive performance. Functional measures included gait speed, expressed as the time to walk 8 m as quickly as possible, and manual dexterity, defined as the time to complete the nine-hole peg test (9HPT) for both hands. Two trials were performed for each of these tasks and mean values were calculated. The degree of extracerebellar involvement was quantified using the Inventory of Non-Ataxia Signs (INAS), which translates into a total INAS count ranging from 0 to 16 [26]. Higher scores indicate more severe extracerebellar pathology. To examine a possible association between cognitive performance and depressive symptoms, all patients were requested to complete the Patient Health Questionnaire-9 (PHQ-9) [27]. Lastly, disease duration and length of the expanded CAG repeat were obtained.

### Statistical analysis

Data are reported as mean and standard deviation (SD), median and interquartile range (IQR), or frequency and percentage, as appropriate. The Shapiro–Wilk test was used to check if continuous variables followed a normal distribution. Total CCAS-S scores of SCA3 patients and controls were compared using an independent samples *t*-test. As individual item scores and the number of failures were non-normally distributed in healthy individuals, Mann–Whitney *U* tests were employed to analyze between-group differences for these variables. Differences in failure percentage per task were evaluated by Fisher’s exact tests. Finally, the discriminative ability of total number of failed tests and total CCAS-S score was expressed as the area under the receiver operating characteristic curve (AUC).

Next, we ascertained whether cognitive deficits in SCA3 patients stand alone or are associated with ataxia severity, extent of extracerebellar involvement, and presence of depressive symptoms. Correlation coefficients were assessed between cognitive performance, as captured by total CCAS-S score, and SARA score, gait speed, manual dexterity, disease duration, length of the expanded CAG repeat, INAS count, and PHQ-9 score. Subsequently, stepwise linear regression analyses (criteria: probability of *F*-to-enter ≤ 0.05, probability of *F*-to-remove ≥ 0.10) were conducted to derive predictors for those neuropsychological tasks that differed significantly between patients and controls. Raw individual test scores served as dependent variables, while SARA score, disease duration, age, and educational level were selected as independent variables. Results are presented as unstandardized (*b*) and standardized (*β*) regression coefficients. Statistical analyses were performed in SPSS Statistics (IBM, version 25). Alpha level of significance was set at 0.05 (two-sided).

## Results

Demographic and clinical characteristics of SCA3 patients and healthy controls matched for age, sex, and educational level are summarized in Table 1.

**Table 1:** Demographic and clinical characteristics of SCA3 patients and healthy controls. Data are presented as mean ± SD or frequency (percentage)

	SCA3 patients	Healthy controls
Age (y)	51.9 ± 10.0	52.9 ± 6.6
Male, *n* (%)	12 (60)	10 (55.6)
Educational level (y)	18.5 ± 4.0	19.7 ± 2.9
Disease duration (y)	8.0 ± 5.4	–
SARA score	11.9 ± 3.9	–
Number of repeats at expanded allele	67.6 ± 3.4	–

### Global performance of the CCAS-S

**Fig. 1:**
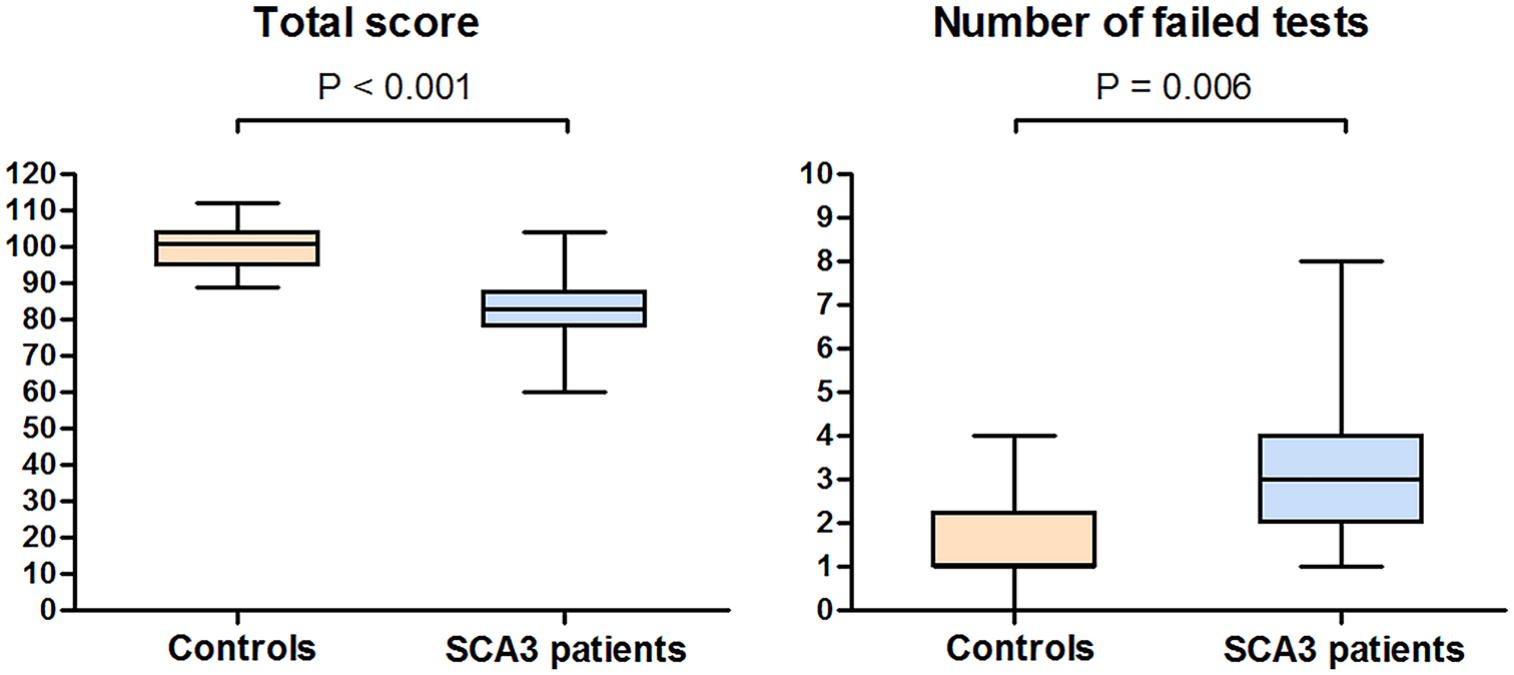
Global performance of the cerebellar cognitive affective syndrome scale in SCA3 patients and healthy controls

**Fig. 2:**
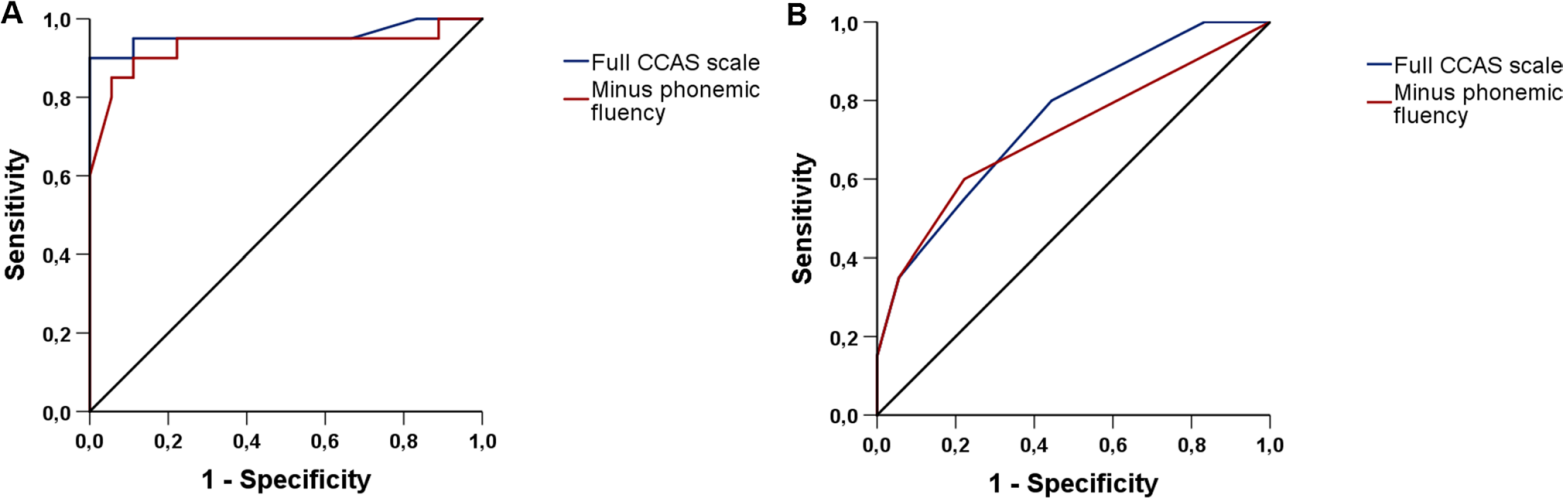
Discriminative ability of total cerebellar cognitive affective syndrome scale score and number of failed tests. Receiver operating characteristic curves illustrate the high discriminative performance of the former (**a**) and the moderate discriminative performance of the latter (**b**). To determine if the phonemic fluency task negatively affected CCAS-S’s overall ability to differentiate SCA3 patients from healthy controls, a separate analysis was conducted without this item (red line)

Significant differences in total CCAS-S score (*t*(36) = 7.13, *p* < 0.001) and number of test failures (*U* = 272, *z* = 2.75, *p* = 0.006) were observed between both groups (Fig. 1). Notably, mean difference in total score between SCA3 mutation carriers (81.9 ± 9.2 points) and healthy controls (100.6 ± 6.6 points) was almost 20 points (Table 2). The distribution of the number of failed tasks in SCA3 patients was as follows: 0 (*n* = 0), 1 (*n* = 4), 2 (*n* = 5), ≥ 3 (*n* = 11). The number of test failures in healthy controls was distributed as follows: 0 (*n* = 3), 1 (*n* = 7), 2 (*n* = 4), ≥ 3 (*n* = 4). AUC values of 0.76 (95% confidence interval [CI] 0.60–0.91) for the number of failed items and 0.96 (95% CI 0.88–1.00) for total CCAS-S score indicate moderate and high discriminative ability, respectively (Fig. 2).

**Table 2:** Performance of the cerebellar cognitive affective syndrome scale in SCA3 patients and healthy controls, expressed as individual test scores and failure percentage per neuropsychological task. Data are presented as median (IQR) or frequency (percentage)

	SCA3 patients	Healthy controls	*P* value
Semantic fluency			
Score	18.0 (14.3–20.8)	25.0 (23.8–26.0)	< 0.001
Individuals failed, *n* (%)	6 (30)	0	0.021
Phonemic fluency			
Score	5.0 (3.0–6.8)	8.0 (7.0–10.3)	< 0.001
Individuals failed, *n* (%)	19 (95)	12 (66.7)	0.038
Category switching			
Score	10.0 (7.3–11.0)	13.5 (11.0–15.0)	< 0.001
Individuals failed, *n* (%)	7 (35)	3 (16.7)	0.28
Digit span forward			
Score	6.0 (5.0–6.8)	6.0 (5.0–6.5)	0.82
Individuals failed, *n* (%)	8 (40)	7 (38.9)	1.0
Digit span backward			
Score	4.0 (3.0–4.0)	4.5 (3.0–5.3)	0.13
Individuals failed, *n* (%)	7 (35)	5 (27.8)	0.74
Cube draw and copy			
Score	13.5 (11.3–15.0)	15.0 (15.0–15.0)	0.003
Individuals failed, *n* (%)	5 (25)	0 (0)	0.048
Incorrect drawing, *n* (%)	10 (50)	1 (5.6)	0.004
Incorrect copying, *n* (%)	5 (50)	0 (0)	1.0
Verbal recall			
Score	13.5 (12.0–15.0)	14.0 (12.0–15.0)	0.55
Individuals failed, *n* (%)	2 (10)	1 (5.6)	1.0
Similarities			
Score	7.0 (7.0–8.0)	8.0 (7.0–8.0)	0.059
Individuals failed, *n* (%)	3 (15)	0 (0)	0.23
Go/no-go			
Score	2.0 (2.0–2.0)	2.0 (2.0–2.0)	0.19
Individuals failed, *n* (%)	1 (5)	0 (0)	1.0
Affect			
Score	5.5 (5.0–6.0)	6.0 (6.0–6.0)	0.001
Individuals failed, *n* (%)	3 (15)	0 (0)	0.23

### Affected neuropsychological domains in SCA3 patients

**Fig. 3:**
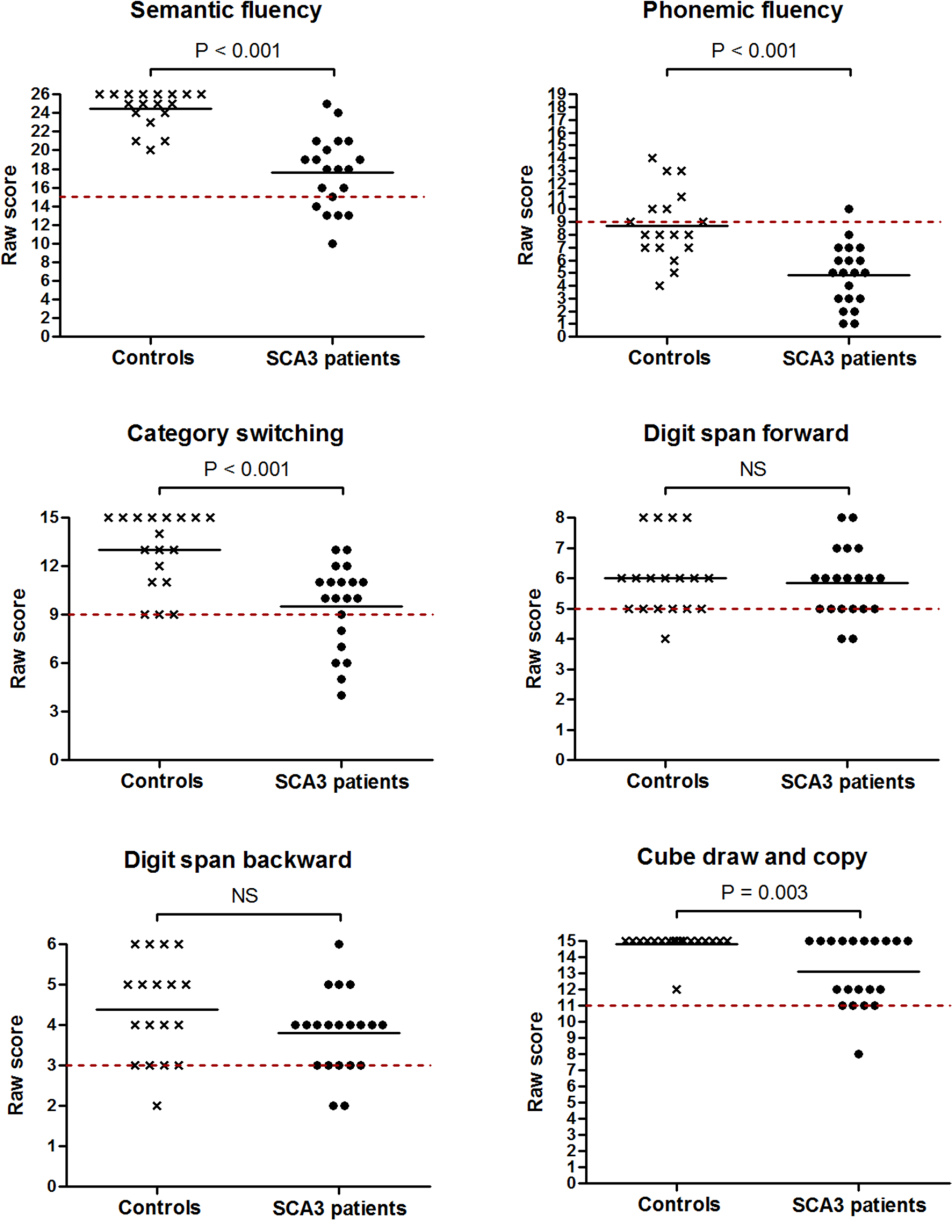

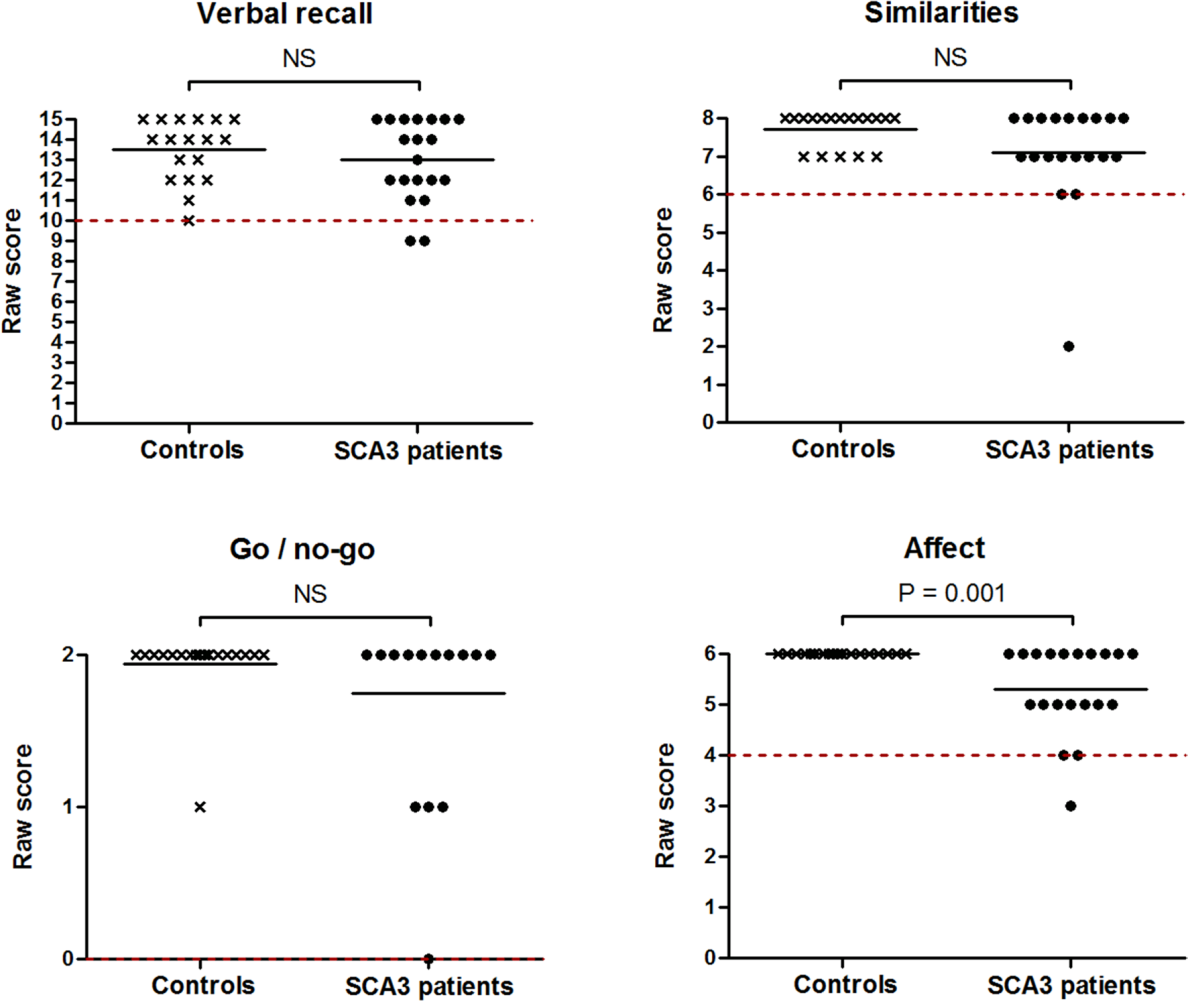
Dot plots displaying individual neuropsychological test scores in SCA3 patients and healthy controls. The dashed red lines represent previously assigned cut-offs that indicate if a participant passed or failed the task [14]

Individual neuropsychological test scores (Fig. 3) showed that SCA3 patients performed worse than controls on semantic fluency (*U* = 19.5, *z* = − 4.73, *p* < 0.001), phonemic fluency (*U* = 48.5, *z* = − 3.87, *p* < 0.001), category switching (*U* = 59.5, *z* = − 3.56, *p* < 0.001), cube drawing and copying (*U* = 97.5, *z* = − 3.02, *p* = 0.003), and affect regulation (*U* = 90.0, *z* = − 3.41, *p* = 0.001). Ten out of twenty patients were unable to correctly draw a cube and fifty percent of this subgroup could not exactly copy a cube. No between-group differences were found in digit span forward, digit span backward, verbal recall, similarities, and go/no-go scores (all *p* > 0.05). SCA3 patients displayed higher failure rates on semantic fluency (*p* = 0.021), phonemic fluency (*p* = 0.038), and cube drawing tasks (*p* = 0.048) than controls. Unexpectedly high proportions of failure were encountered in healthy individuals on phonemic fluency (66.7%), digit span forward (38.9%), and digit span backward items (27.8%). To determine if the phonemic fluency task negatively affected CCAS-S’s overall ability to discriminate between patients and controls, we subsequently recomputed AUCs for the number of failed tests and total CCAS-S score when this item was left out. The resultant decrease in AUC values from 0.76 to 0.72 (95% CI 0.55–0.88) and from 0.96 to 0.93 (95% CI 0.84–1.00) indicates that the phonemic fluency item positively contributed to the distinction between both groups.

### Associations between cognitive performance and disease severity measures

**Fig. 4:**
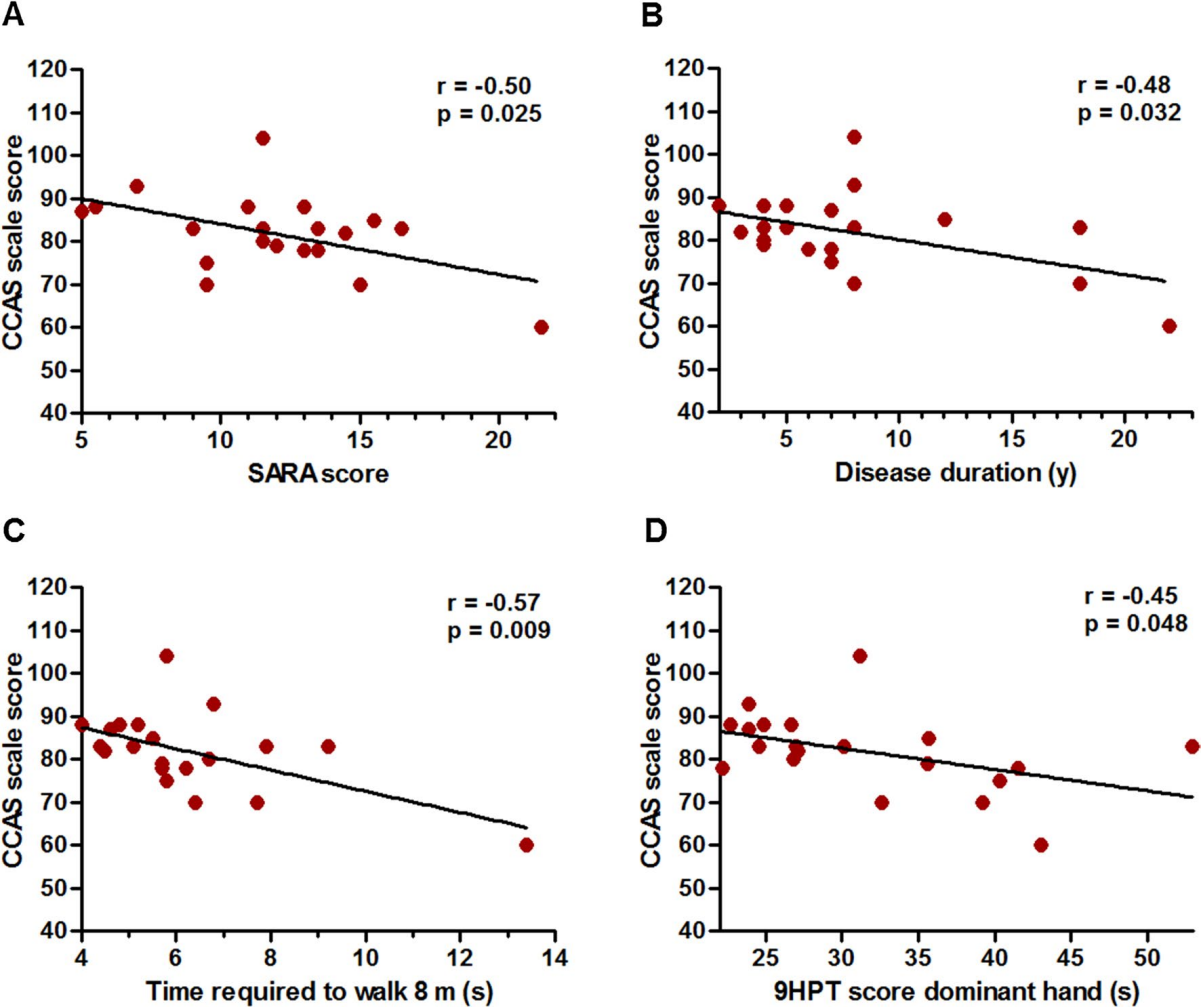
Associations between total cerebellar cognitive affective syndrome (CCAS) scale score and relevant disease severity measures in SCA3 patients. *SARA* Scale for the Assessment and Rating of Ataxia; *9HPT* nine-hole peg test

As shown in Fig. 4b, total CCAS-S score declined with increasing disease duration (*r* = − 0.48, *p* = 0.032). Although we do not have empirical data to accurately predict the onset of cognitive deficits in SCA3 patients, extrapolation of the fitted regression line would reach the mean value of healthy controls at minus 15 years disease duration, corresponding to a mean age of 28.9 years. Albeit speculative, this finding may suggest an onset even before the emergence of motor symptoms. Furthermore, inverse associations were observed between total CCAS-S score and motor symptom severity (Fig. 4), expressed by SARA score (*r* = − 0.50, *p* = 0.025), time required to walk 8 m (*r* = − 0.57, *p* = 0.009), and dominant hand 9HPT performance (*r* = – 0.45, *p* = 0.048). Conversely, there were no correlations between total CCAS-S score and INAS count, PHQ-9 score, and repeat length of the expanded allele (all *p* > 0.2). We additionally determined associations between affective dysfunction, as rated in the final CCAS-S item, and total CCAS-S score, ataxia severity, gait speed, and manual dexterity. These analyses revealed that affective changes are related to cognitive deficits (*r* = 0.63, *p* = 0.003) but not to motor symptom severity (SARA score: *r* = − 0.16, *p* = 0.51; gait speed: *r* = − 0.24, *p* = 0.32; dominant hand 9HPT performance: *r* = − 0.31, *p* = 0.19; non-dominant hand 9HPT performance: *r* = − 0.07, *p* = 0.77).

### Predictors of cognitive deficits in SCA3 patients

To identify factors that are independently associated with cognitive impairments in SCA3 patients, we performed stepwise linear regression analyses with disease duration, SARA score, age, and educational level as predictor variables. We found that disease duration (*b* = − 0.67 [SE = 0.30], *β* = – 0.39, *p* = 0.04) and educational level (*b* = 1.17 [SE = 0.41], *β* = 0.51, *p* = 0.01) independently affected total CCAS-S score. Together, these variables explained 42% of the variance in total score, as reflected by the adjusted R^2^ value. Similar models with individual item scores as dependent variables showed that semantic fluency was predicted by SARA score (*b* = − 0.48 [SE = 0.20], *β* = – 0.48, *p* = 0.032), category switching by disease duration (*b* = − 0.34 [SE = 0.08], *β* = – 0.70, *p* = 0.001), cube drawing and copying by educational level (*b* = 0.26 [SE = 0.09], *β* = 0.49, *p* = 0.009) and disease duration (*b* = − 0.19 [SE = 0.07], *β* = – 0.47, *p* = 0.011), affect by educational level (*b* = 0.15 [SE = 0.04], *β* = 0.71, *p* < 0.001), and phonemic fluency by none of the independent variables. Educational level (*b* = − 0.18 [SE = 0.09], *β* = – 0.40, *p* = 0.048) and age (*b* = 0.079 [SE = 0.03], *β* = 0.43, *p* = 0.034) affected the number of failed tests significantly.

## Discussion

This study examined the performance of the CCAS-S in a cohort of SCA3 patients and yielded three main findings. First, essentially all SCA3 patients displayed some form of cognitive impairment, even if they were only mildly to moderately affected in the motor domain, and total CCAS-S score reliably differentiated them from healthy controls. Second, our data confirm the presence of deficits in executive functions, linguistic processing, visuospatial and visuoconstructive abilities, and affect regulation in SCA3. Third, total CCAS-S score was related to disease duration and relevant ataxia severity indices, such as SARA score, gait speed, and manual dexterity. Conversely, no association was found with the extent of extracerebellar involvement, as measured by total INAS count, and with PHQ-9 score.

### Cognitive impairments in SCA3 patients

Cognitive deficits are nowadays widely acknowledged to constitute an integral part of cerebellar diseases, particularly if the posterior lobules VI, VIIA Crus I/II, VIIB, and IX, and their reciprocal connections with cortical association areas and limbic regions are implicated [6, 7, 11–13, 28]. Here, we demonstrate significantly lower scores on semantic fluency, phonemic fluency, category switching, cube drawing, and affect regulation items in SCA3 patients compared to healthy controls. Combined with the absence of between-group differences in immediate and delayed verbal recall, our findings may point to a “cerebellar cognitive phenotype” [6, 12, 14]. Autopsy studies in SCA3 patients typically reveal severe neuronal loss in the dentate nuclei, which act as important relay stations between cerebellar and cerebral cortices, along with relative sparing of the cerebellar cortex [29]. Degeneration of these output channels directly disrupts cerebello-cerebral connectivity, which may be among the mechanisms underlying cognitive deterioration in our patients. This hypothesis is strengthened by results from Tedesco and colleagues who showed that involvement of deep nuclei in individuals with focal cerebellar lesions negatively predicted cognitive performance [30]. Furthermore, the deep nuclei and cerebellar outflow pathway were found to be significantly more injured in children who developed CCAS after resection of a cerebellar tumor than a matched comparison group without CCAS [31]. Neuropathology of SCA3 is complex, however, and extends beyond the cerebellum in most cases. Dysfunction of basal ganglia-thalamocortical loops and cerebral cortical damage have also been reported and may contribute to cognitive deficits in SCA3 patients [18, 29, 32–35].

### Performance of the CCAS-S: total score and number of failed tests

We observed a particularly high discriminative ability of total CCAS-S score in our study population. Despite the relatively low number of patients, the large differences with healthy controls would support its use for cognitive screening purposes in SCA3 cohorts. In addition, the independent inverse association between total CCAS-S score and disease duration may encourage the application of this measure in the monitoring of cognitive performance over time. Long-term follow-up results are currently lacking due to the recent publication of the scale, but our cross-sectional data suggest a decrease of 0.67 points per year. The association with disease duration and the observation that differences with healthy controls were already demonstrated in mildly affected patients raise the question at which point in the disease process total CCAS-S score starts to diverge. Although our study only included individuals with manifest ataxia, extrapolation of the regression line leads to the hypothesis that objectifiable differences may be detected prior to the onset of motor symptoms. Interestingly, disturbances in delay eyeblink conditioning, an associative learning paradigm that critically depends on the integrity of lobule VI and the interposed nuclei, were already found in preclinical SCA3 mutation carriers [36]. Moreover, decreased phonemic fluency and executive dysfunction have been described during the prodromal stage of SCA2 and were inversely correlated with estimated time to ataxia onset [37].

In line with the between-group difference in total score, SCA3 patients also exhibited a higher number of failed tests. Failures occurred more often on semantic fluency, phonemic fluency, and cube drawing compared to controls. Similar to a recent study involving nineteen Belgian individuals with Friedreich ataxia, all SCA3 mutation carriers failed at least one CCAS-S item [38]. The high sensitivity of the CCAS-S to detect cognitive impairment in cerebellar diseases was further supported by an evaluation of ten individuals with late-onset GM2 gangliosidosis [39]. Whereas subjective cognitive complaints had been reported by only one patient or caregiver, five out of ten met criteria for definite CCAS, two for probable CCAS, and two for possible CCAS, sometimes even in the absence of brain MRI abnormalities. A surprisingly high rate of failed tests was encountered in controls without interfering disease or drug use, yielding a moderate AUC value. Regression analyses excluded age and educational level as possible confounders. The specific cause of controls’ failure on digit span forward and backward tasks remains elusive although we speculate that the allowance of one trial per digit sequence rather than two or more might have played a role. Additionally, the letter F in the phonemic fluency item proved to be difficult for a number of healthy individuals. Nonetheless, controls’ scores were nearly double those of patients and a separate AUC analysis revealed a positive contribution of this item to the overall discriminative ability of the CCAS-S. Selection of a more commonly used letter for this task may have lowered the number of failures, though possibly at the expense of less obvious contrasts between groups.

Besides the previously mentioned investigations in Friedreich ataxia and GM2 gangliosidosis, our study was the third to apply the CCAS-S in an etiologically homogeneous cohort of ataxia patients. To the best of our knowledge, there is only one other study that examined the performance of this scale in eleven Belgian patients with heterogeneous pathologies confined to the cerebellum, including two individuals with essential tremor. [25] No significant differences were found between patients and controls except in digit span forward and category switching tasks, and the possibility of atypical cerebellar dysfunction was suggested as an explanation. This observation calls for further investigations into the CCAS-S and its dynamics over time in etiologically homogeneous patient cohorts, preferably in combination with more detailed neuropsychological assessments.

### Associations between cognitive dysfunction and motor symptom severity

It has been postulated that cerebellar cognitive and motor functions are subserved by distinct regions of the cerebellar cortex [7, 8, 13]. We found significant associations between total CCAS-S score and SARA score, walking speed, and 9HPT performance, which at first glance appears to be at odds with this sensorimotor–cognitive dichotomy hypothesis. However, in light of the multifocal neuronal loss that characterizes a degenerative disorder such as SCA3, it is not unlikely that different regions subserving distinct functions are simultaneously affected. Alternatively, these cognitive–motor correlations may indicate involvement of a shared critical structure that incorporates functionally segregated but closely positioned components of both networks. Indeed, the dentate nuclei contain both (dorsal) motor and (ventral) non-motor domains, and, as outlined above, are usually severely affected in SCA3 [1, 29, 40]. Intriguingly, a similar parallel between ataxia severity and cognitive impairment, as determined using the CCAS-S, was recently described in patients with Friedreich ataxia, which may also be attributable to dentate nucleus pathology [38]. The absence of structural brain scans in our patients at the time of testing precludes analyses on the contributions of cerebellar subregions to cognitive and motor performance. Regardless of the precise underlying mechanism, total CCAS-S score seems to be a marker that reflects general disease progression in SCA3. Finally, in this context, it is interesting to note that affective dysregulation was closely associated with cognitive deficits but unrelated to motor impairment.

## Conclusion

The CCAS-S differentiates SCA3 patients from healthy controls, detects neuropsychological deficits early in the disease course, and correlates with relevant ataxia severity measures. In addition to neuropsychological screening, our findings lend indirect support to its use in cognitive follow-up evaluations. Longitudinal examinations are required to test the assumptions we made based on cross-sectional data, most notably the estimated yearly decline in total CCAS-S score. Furthermore, important information concerning the onset of cognitive deficits could be derived from the application of this instrument in preclinical SCA mutation carriers.
